# Jejunal Interposition with Overlap Esophago-Jejunal Anastomosis for an Esophageal Stricture due to Repeated Endoscopic Dilation for Esophageal Achalasia: A Case Report

**DOI:** 10.70352/scrj.cr.25-0033

**Published:** 2025-06-18

**Authors:** Yasuto Suzuki, Shinsuke Takeno, Fumiaki Kawano, Kousei Tashiro, Makoto Ikenoue, Kazunosuke Yamada, Atsushi Nanashima

**Affiliations:** Department of Surgery, Faculty of Medicine, University of Miyazaki, Miyazaki, Miyazaki Japan

**Keywords:** esophageal achalasia, esophageal stricture, jejunal interposition, overlap anastomosis

## Abstract

**INTRODUCTION:**

Achalasia is a primary esophageal motility disorder of unknown origin. The clinical manifestations are caused by the loss of peristalsis of the esophagus and functional obstruction at the esophagogastric junction. There are several treatment strategies for esophageal achalasia, such as medications, endoscopic treatment, and surgery. The successful treatment of a case of jejunal interposition surgery with overlap esophago-jejunal anastomosis for an esophageal stricture due to repeated endoscopic dilation for esophageal achalasia is reported.

**CASE PRESENTATION:**

The patient was a 67-year-old man who was diagnosed with esophageal achalasia 13 years earlier. Partial esophagectomy of the portion with the stricture and esophago-jejunal anastomosis using the overlap method were performed for the esophageal stricture due to rupture during endoscopic balloon dilatation. The patient’s postoperative recovery was unremarkable, and the dysphagia due to esophageal stricture disappeared.

**CONCLUSIONS:**

The overlap technique in esophago-jejunal anastomosis after partial esophagectomy was very effective for an esophageal stricture in a patient with achalasia because it made possible the additional resection of endoluminal muscle.

## Abbreviations

LES
: lower esophageal sphincter
POEM
: per oral endoscopic myotomy

## INTRODUCTION

Esophageal achalasia is a rare disease, with an incidence of 0.5–1.0 patients per 100000 population a year. Esophageal achalasia is defined as an esophageal transit disorder caused by inadequate relaxation of the lower esophageal diverticulum and abnormal dilation of the esophagus.^1)^

Pharmacotherapy relaxes the LES and reduces LES pressure by 47–63%, and it is the treatment of choice for mild cases that are not amenable to endoscopic therapy.^2)^

Endoscopic treatment includes balloon dilation and POEM. Endoscopic balloon dilatation is a treatment to decrease LES pressure by stretching and tearing the esophageal endoluminal muscle of the LES. POEM is Heller’s myotomy technique performed by endoscopy. In POEM, the length of the myotomy incision is relatively flexible, and the patient does not require laparotomy. POEM is an effective treatment for esophageal achalasia, and its use has increased rapidly.^3)^

However, endoscopic treatment cannot be performed in some patients with severe fibrosis caused by previous multiple treatments and complications of endoscopic treatment.^4)^

Although Roux was the first surgeon to replace the esophagus with the jejunum in 1907,^5)^ Longmire was the first to describe a long-segment jejunal interposition with microvascular augmentation.^6)^ The complexity of the operation precluded widespread use despite these early reports demonstrating the technical feasibility of the augmented blood supply to the long-segment pedicled jejunal interposition.^7)^

The overlap technique is a classic technique using a linear stapler for anastomosis, which was proposed by Inaba et al. in 2010 as a reconstruction method for the marginal esophageal jejunal anastomosis after total gastrectomy.^8)^ In 2013, Nagai et al. reported an improved overlap technique,^9)^ and in 2017, Huang et al. proposed an anastomosis technique called the posterior jejunal transection overlap.^10)^

In this report, surgical treatment using jejunal interposition with overlap esophago-jejunal anastomosis for an esophageal stricture after partial esophagectomy due to repeated endoscopic dilation for a patient with esophageal achalasia with a benign esophageal stricture is described.

## CASE PRESENTATION

A 67-year-old man was diagnosed with an esophageal stricture 13 years earlier by esophagogastroduodenoscopy. The esophageal stricture lesion began to worsen at 60 years of age, and endoscopic balloon dilatation was performed 16 times between the ages of 60 and 67 years. After dilatation three times, mediastinal emphysema was observed, and a nasogastric tube decompression and therapeutic fasting was required.

**Fig. 1:**
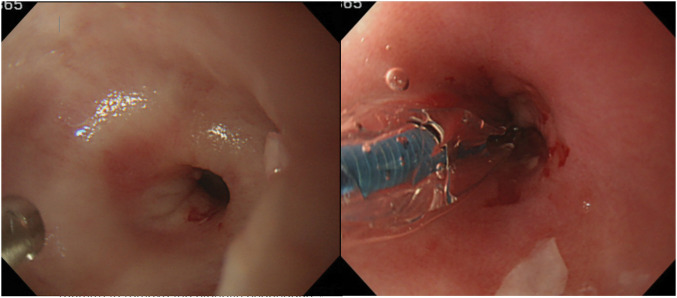
Endoscopic dilatation performed before surgery. Stenosis in the lower esophagus, through which it is difficult to pass the endoscope. The esophagus is dilated from 8 mm to 9 mm, but endoscopic passage is difficult even after dilatation.

**Fig. 2:**
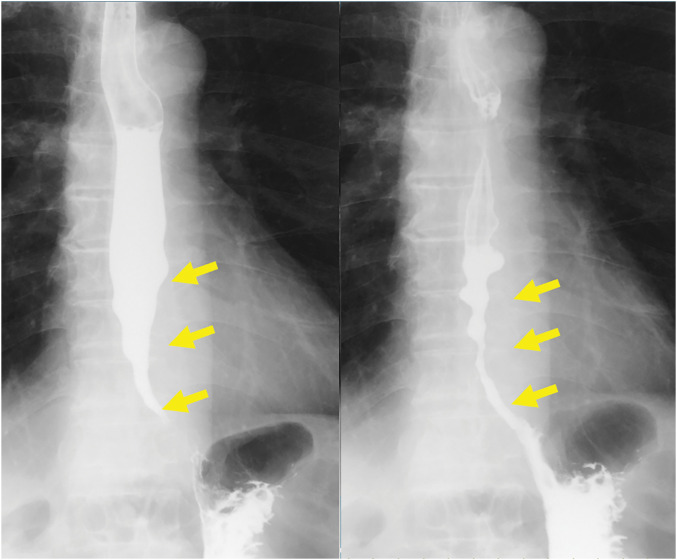
Esophagogram performed before surgery. Barium stagnation in the esophagus with a smooth narrow appearance (bird’s beak sign) is seen (yellow arrows). Straight type findings with no esophageal meandering. Insufficient dilatation and irregular peristalsis extend into the middle esophagus.

An esophagogram showed inadequate dilatation and irregular peristalsis extending into the middle esophagus (**Fig. 1**). Gastroscopy showed stenosis in the lower esophagus, and the endoscope could not pass through the stenotic lesion. Although endoscopic dilatation was performed using a 9-mm balloon, there was no effect on the stenotic lesion (**Fig. 2**).

POEM was considered, but submucosal tunneling was difficult because of the stricture in the lower esophagus, and the risk of mediastinitis after POEM was considered high. Heller-Dor surgery was ruled out because it was thought to be difficult to perform a myotomy from the adventitia side due to the strong scar. It was determined that resection of the scarred and narrowed lower esophagus was necessary.

At surgery, the abdomen was opened through a median upper abdominal incision approximately 8 cm from the xiphoid process. A lesser omentum was opened to reach the esophageal hiatus through the right leg and taped to the abdominal esophagus. The transverse ligament was separated, and the esophageal hiatus was opened. The vagus nerve was transected during esophageal taping. The lower esophagus was dissected in the inferior mediastinum, and an upper gastrointestinal endoscope was used to identify the scarred stenotic area. The lower mediastinum showed strong adhesions between the surrounding tissue and the lower esophagus, which was thought to be due to endoscopic treatment and mediastinal emphysema. The lower esophagus was dissected using a stapling device (60 mm) to remove the stenotic esophagus.

**Fig. 3:**
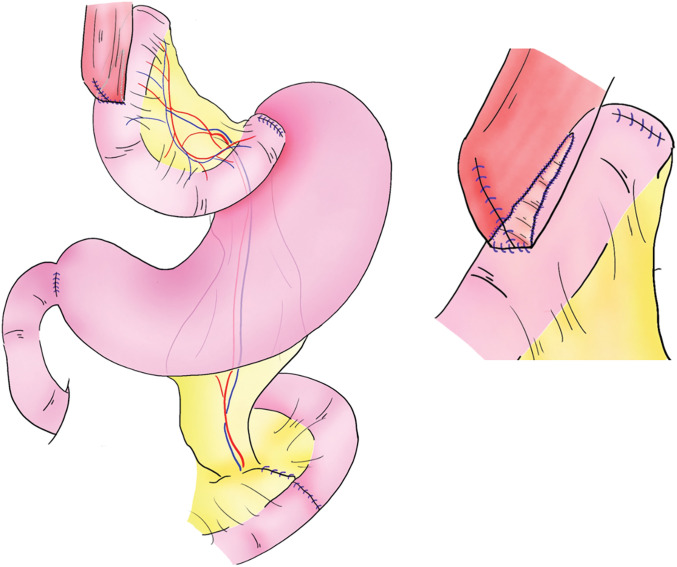
The second jejunal artery is dissected, a graft created, and the graft raised dorsally. The esophageal transection and the elevated jejunum are perforated and anastomosed laterally using the overlap technique, and the common hole is sutured with 2 layers of hand stitches. A 60-mm automatic suturing device is used. A gastric and jejunal anastomosis is also performed using the overlap method to prevent stenosis, and pyloroplasty using the HeinekeMikulicz method is added to prevent reflux esophagitis.

The second jejunal artery was sacrificed to create a jejunal graft. The jejunal graft was pulled-up via the retro-mesenteric and stomach route. The esophago-jejunal anastomosis was performed ventrally with the overlap technique using a stapling device (60 mm). The common hole was closed with an Albert Lembert suture. The gastro-jejunal anastomosis was created by the overlap method using a stapling device (60 mm) at a distance of more than 15 cm from the esophago-jejunal anastomosis. The pulled-up jejunal graft was suture-fixed to the left and right diaphragmatic legs with one stitch each, and pyloroplasty was performed by the HeinekeMikulicz technique (**Fig. 3**).

**Fig. 4:**
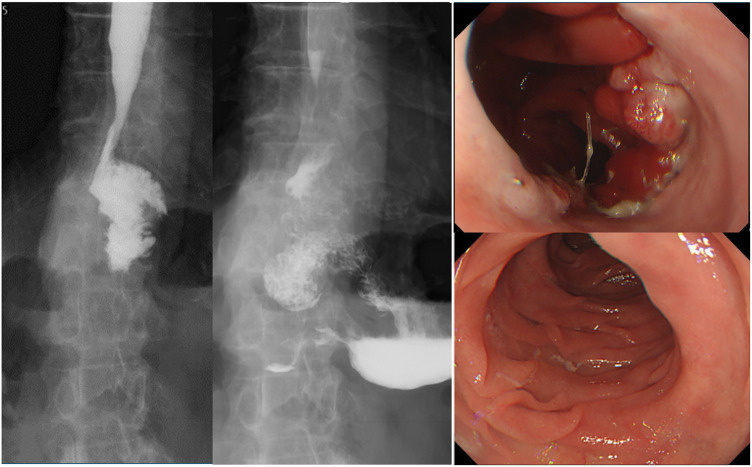
Esophagography shows no obstruction to passage of the esophago-jejunal anastomosis. Endoscopy shows no stenosis at the esophago-jejunal anastomosis, and endoscopic passage is possible.

Esophagography was performed 7 days after surgery and showed no stricture at the esophago-jejuno-gastric suture, and the patient started eating. Esophagography and endoscopy were performed on postoperative days 14 and 16, and no stenosis or reflux esophagitis was observed. The patient was discharged on postoperative day 18 with no anastomotic stenosis, anastomotic failure, and symptoms of delayed gastric empty (**Fig. 4**). After discharge from the hospital, the patient was free of symptoms of stenosis and had good food intake.

## DISCUSSION

In this case, after repeated endoscopic balloon dilatations, the esophageal stricture became so severe that endoscopic treatment, including myotomy, was difficult. Surgical treatment of the esophageal stricture was necessary, and resection of the esophageal stricture and reconstruction with jejunal interposition with overlap esophago-jejunal anastomosis for the esophageal stricture was very effective in this case.

Balloon dilatation and POEM treatment are widely used and effective for esophageal achalasia. However, complications such as perforation and repeated treatment have been reported to cause narrowing with esophageal scar, even with prophylactic measures such as injection of medication.^11)^ There is no established treatment for esophageal stricture after multiple endoscopic procedures. Severe scarring makes it difficult to create a submucosal tunnel and increases the risk of complications such as mediastinitis by endoscopic treatment. In addition, repeated treatment may result in extensive narrowing by scar, which requires surgical treatment.

In cases of recurrence after myotomy, surgical myotomy often only allows lateral myotomy after an anterior approach. In POEM, a myotomy is generally attempted in the opposite direction of the first myotomy.^12)^ Repeated balloon dilatations are expected to cause circumferential scar stenosis centered on the dilated area. The present patient had undergone 17 balloon dilatations, and the circumferential scarring made myotomy difficult. In the case of a condition caused by esophageal achalasia, simple resection of the esophageal scar alone may result in residual hypercontractility of the esophageal endoluminal muscle, which could lead to a postoperative passage disorder.

For anastomosis of the esophagus and jejunum after partial esophagectomy, there is an overlap technique using a straight stapler and reconstruction using a circular stapler. A comparison of the overlap method and the circular stapler anastomosis in esophago-jejunal anastomosis in total gastrectomy has also been reported; it has been reported that anastomotic stenosis is less than with circular stapler anastomosis.^13,14)^ The use of straight staples for anastomosis has been reported to create a wide lumen diameter (30 mm or greater), leading to a decreased risk of stenosis.^15)^ In the present case, a 60-mm-long automatic suture was used for lateral anastomosis of the esophagus and jejunum. By using a 60-mm-long automatic suture, it was possible to add a 60-mm-long incision of the esophageal endoluminal muscle in addition to the length of the resected scar stenosis. This would be surgical treatment for esophageal achalasia by myotomy in addition to prevent postoperative stenosis and obstruction of passage.

The jejunum possesses all the physiological characteristics of a sphincter by virtue of its inherent rhythm and isoperistalsis, its lack of fatigability, and its intestinal reaction to a bolus.^16)^ It is also apparent that the jejunal segment is more resistant to acid reflux than either the stomach or duodenum.^16)^ In addition, peristalsis of the jejunum can be expected to prevent reflux symptoms. The remaining stomach has a transected vagus nerve, which can cause gastric atony, stagnation and retention of gastric contents, and reflux into the oral side.^17)^ There are methods of esophageal and gastric reconstruction that involve a long-axis incision in the esophagus, such as the SOFY procedure, but postoperative reflux esophagitis is a concern.^18)^ Taking advantage of the characteristics of the jejunum, a sufficiently long jejunal interposition is expected to reduce the risk of postoperative reflux esophagitis. To prevent reflux esophagitis, it is important not only to use the jejunum as a reconstructive organ, but also to add pyloroplasty by the HeinekeMikulicz method to prevent gastric stagnation.

There have been reports of jejunal interposition in recurrent stenosis after Heller myotomy, and Keller et al. reported 17 cases of distal benign esophageal stenosis treated with jejunal replacement. In all, 10 of the 17 cases of recurrence after Heller surgery included myotomy or dilation. In the present case, few postoperative complications were encountered. The most frequent problem directly related to the surgery was duodenal obstruction due to the elevated mesenteric pedicle.^19)^

For surgical procedures using the jejunum, the double-tract method can be considered as a reconstructive method. Lu et al. compared the results of jejunal interposition and the double-tract method as a method of reconstruction for proximal gastrectomy. In this report, surgical time in the jejunal interposition group was significantly longer than in the double-tract group. However, there was no advantage in the incidence of reflux esophagitis, anastomotic complications (including anastomotic stricture, anastomotic bleeding, and anastomotic leakage), or overall complications. Several indices reflecting nutritional status, such as body weight, hemoglobin, total protein, serum albumin, and vitamin B12, were reported to be higher in the jejunal interposition group than in the double-tract group.^20)^

In Nomura’s studies in 2014 and 2018, apart from the above-mentioned indicators, they also compared the absorption curves of acetaminophen, insulin levels, and blood glucose levels of the two groups of patients after a meal. In the double-tract group, the increases in the blood sugar level at 30 and 60 minutes were more gradual than in the jejunal interposition group. This report also suggests that jejunal interposition is considered ideal for function-preserving gastrectomy, but the double-tract approach may be more appropriate for patients with impaired glucose tolerance.^21,22)^

The present patient had no history of diabetes mellitus, and no abnormal blood glucose levels were noted on preoperative examination. Therefore, there was no need to consider postoperative glycemic control. From a physiological functional reconstruction and nutritional standpoint, jejunostomy was considered appropriate for the present patient. Although the surgery took approximately 6 hours, it was performed safely without any problems during the procedure. For the esophago-jejunal anastomosis, the overlap technique was used, and the esophageal meatus was also incised to secure the anastomotic lumen and prevent anastomotic stenosis. In addition, the jejunal interposition as a reconstructive organ prevented reflux esophagitis. This resulted in a good postoperative course for the patient, with no food deprivation or suture defects. It was considered possible to shorten the operation time by establishing a surgical technique and procedure.

## CONCLUSIONS

The overlap technique in esophago-jejunal anastomosis after partial esophagectomy was very effective for an esophageal stricture in a patient with achalasia because it made possible the additional resection of endoluminal muscle.

Although the surgical technique for esophageal interposition is more complicated, it can be used for any stenosis, and the risk of postoperative stenosis can be reduced.

## ACKNOWLEDGMENTS

The authors thank FORTE Science Communications (https://www.forte-science.co.jp/) for English language editing.

## DECLARATIONS

### Funding

None.

### Authors’ contributions

All of the authors contributed to the diagnosis and treatment of the patient.

YS contributed to the drafting of the manuscript.

ST and KY edited the manuscript.

ST and KY supervised and gave final approval of the manuscript.

All authors read and approved the final manuscript.

### Availability of data and material

Data sharing is not applicable to this article, as no datasets were generated or analyzed during the current study.

### Ethics approval and consent to participate

Not applicable.

### Consent for publication

The patient provided written informed consent for the publication of this paper.

### Conflict of interest

The authors declare no conflicts of interest in association with the present study.
